# Lung adenocarcinoma with KRAS-Q61H: clinicopathologic features, diagnostics, and the evolving treatment landscape

**DOI:** 10.3389/fonc.2026.1771549

**Published:** 2026-02-11

**Authors:** Ioannis Serafimidis

**Affiliations:** Center of Basic Research, Biomedical Research Foundation of the Academy of Athens, Athens, Greece

**Keywords:** KRAS-Q61H, lung adenocarcinoma, MAPK signaling, peritoneal metastasis, precision oncology, TP53 co-mutations

## Abstract

KRAS is one of the most frequently mutated oncogenes in lung adenocarcinoma (LUAD), with the KRAS-Q61H mutation representing a rare but biologically distinct subgroup. Although KRAS-Q61H is associated with more aggressive clinical behavior, including advanced-stage disease at diagnosis and atypical metastatic spread, its molecular characteristics are not fully understood. This mutation preferentially activates the RAF-MEK-ERK pathway and has been shown to exhibit relative independence from upstream signaling factors like SHP2 and SOS1, distinguishing it from other KRAS mutations. KRAS-Q61H is frequently co-mutated with TP53, and this co-alteration has been linked to increased genomic instability, invasion, and metastatic potential, particularly peritoneal dissemination, which is a feature shared with other cancers harboring KRAS-Q61H, such as pancreatic ductal adenocarcinoma (PDAC) and colorectal cancer (CRC). Comprehensive molecular profiling, including next-generation sequencing (NGS) and plasma-based liquid biopsy, is critical for the early detection of KRAS-Q61H and its co-mutations, enabling more personalized treatment approaches. Despite the lack of approved allele-specific therapies, emerging treatment strategies targeting the MAPK pathway, SHP2, SOS1, and pan-KRAS inhibitors offer hope for more effective management. This review provides an in-depth analysis of the clinical, molecular, and therapeutic aspects of KRAS-Q61H LUAD, with a particular focus on its metastatic behavior, the impact of co-mutations, and the urgent need for molecular profiling in guiding treatment decisions.

## Introduction

**Figure 1:**
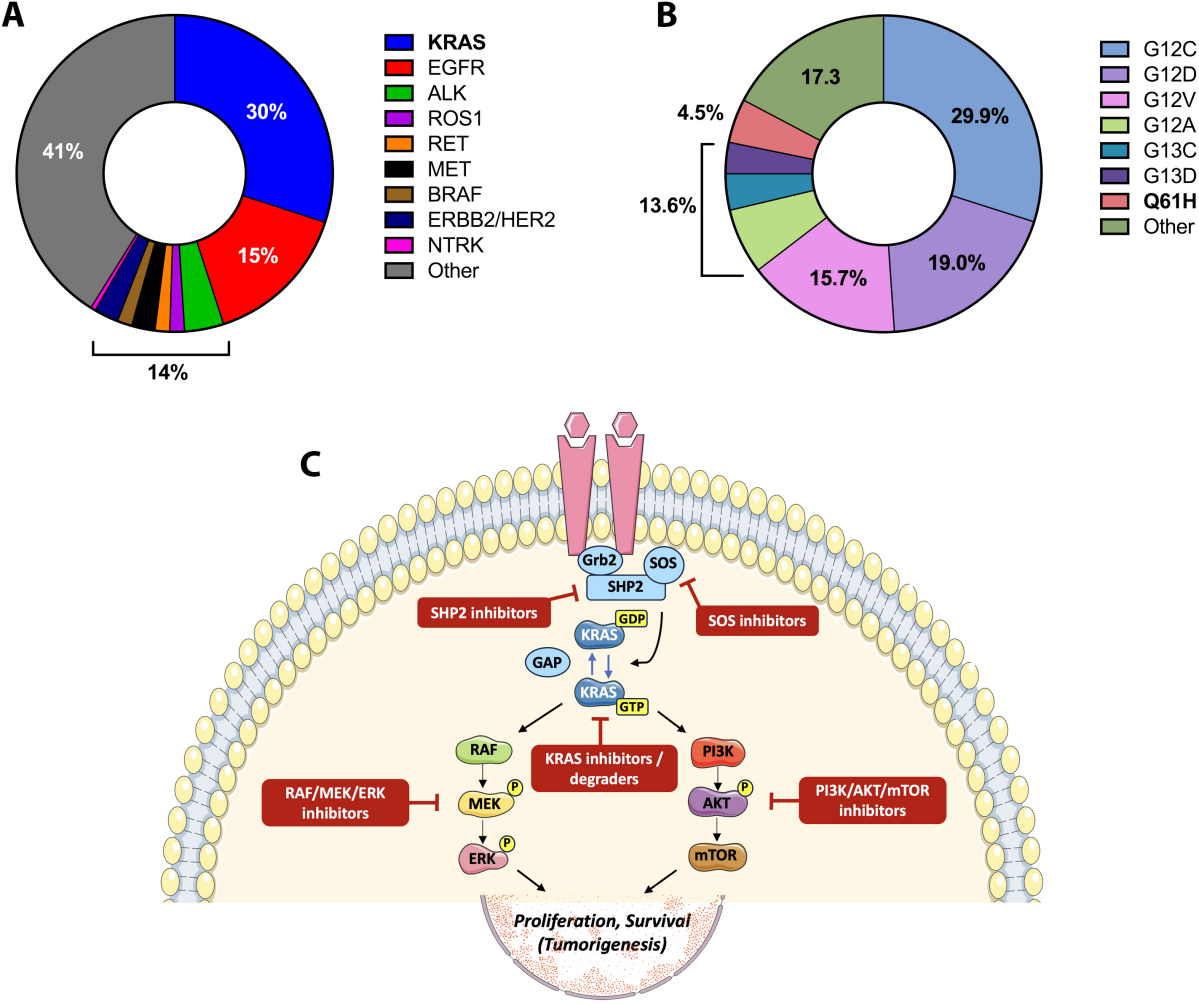
Molecular drivers and therapeutic context of KRAS^Q61H^ lung adenocarcinoma. **(A)** Distribution of major oncogenic drivers in lung adenocarcinoma (LUAD) across Western cohorts, highlighting the predominance of KRAS mutations relative to other actionable alterations; **(B)** Relative distribution of KRAS mutation subtypes in LUAD, illustrating the predominance of codon 12 variants alongside less frequent non-G12 alterations, including codon 61 mutations such as KRAS^Q61H^; **(C)** Schematic representation of KRAS signaling and therapeutic intervention points relevant to KRAS^Q61H^-mutant LUAD. The diagram illustrates guanine nucleotide cycling of KRAS, downstream activation of the RAF-MEK-ERK and PI3K-AKT-mTOR pathways, and current or investigational therapeutic strategies, including SHP2 and SOS1 inhibitors, MAPK pathway inhibitors, PI3K/AKT/mTOR inhibitors, and pan-KRAS inhibitors or degraders.

Lung adenocarcinoma is the most common histologic subtype of non-small cell lung cancer (NSCLC) and is characterized by a complex landscape of oncogenic drivers. Among these, KRAS mutations occupy a prominent position, accounting for more than one-quarter of cases worldwide (1, 2) (**Figure 1A**). The therapeutic history of KRAS has long been marked by frustration, with the protein regarded as a canonical “undruggable” target. This perception shifted with the advent of covalent KRAS^G12C^ inhibitors, which validated KRAS as a tractable target in the clinic (3). Yet the enthusiasm around G12C has highlighted, rather than resolved, the unmet need for patients whose tumors carry non-G12C mutations.

Within this broader group, codon 61 substitutions represent a minority but biologically intriguing subset. Q61H, the most frequent among them, constitutes less than 5% of all KRAS mutations (**Figure 1B**) and just under 1% of all LUAD cases (4, 5). Despite its rarity, Q61H is notable because of its unique biochemical features and the absence of any allele-specific treatment strategies. Moreover, retrospective evidence suggests that the prognosis of Q61H carriers may differ from patients with more common KRAS alleles (6). In the following sections, we review the epidemiology, clinicopathologic associations, molecular characteristics, diagnostic considerations, and therapeutic strategies for KRAS^Q61H^ LUAD, with a particular focus on metastatic behavior, and we conclude by outlining future priorities for clinical development.

## Clinicopathologic features of KRAS^Q61H^ lung adenocarcinoma

Large-scale sequencing studies consistently place KRAS mutations in approximately 20–30% of lung adenocarcinomas, with codon 12 variants predominating and codon 61 alterations remaining uncommon (1, 4). Within this latter group, KRAS^Q61H^ represents the most frequently observed codon 61 substitution, accounting for roughly 4–6% of KRAS-mutant LUAD across Western and Asian cohorts (4–6). Although numerically rare, this mutation defines a reproducible clinicopathologic entity whose features appear to diverge from those of more common KRAS alleles.

Clinically, LUAD harboring KRAS^Q61H^ shares several features with adenocarcinomas of other organs carrying the same mutation, particularly pancreatic ductal adenocarcinoma (PDAC) and colorectal cancer (CRC). Across these tumor types, KRAS^Q61H^ is consistently associated with aggressive clinical behavior, early metastatic dissemination, and inferior outcomes compared with codon 12 variants (7, 8). In LUAD, retrospective series suggest shorter overall survival for patients with KRAS^Q61H^ compared with those harboring KRAS^G12C^ or KRAS^G12D^ (6), echoing observations in PDAC where codon 61 mutations correlate with rapid disease progression and poor response to standard chemotherapy (7, 9).

From a pathological standpoint, KRAS^Q61H^ LUAD shows enrichment in invasive mucinous adenocarcinoma (IMA), a subtype characterized by abundant intracytoplasmic mucin production, aerogenous spread, and a propensity for diffuse and multifocal disease (10). Similar mucinous differentiation is frequently observed in KRAS^Q61H^-mutant CRC and pancreatic tumors, suggesting that this allele may favor transcriptional programs promoting mucin secretion and altered cell–cell adhesion (8, 11). These shared histologic features reinforce the concept that KRAS^Q61H^ drives convergent phenotypes across epithelial adenocarcinomas despite distinct tissue contexts.

One of the most striking parallels between LUAD and gastrointestinal adenocarcinomas bearing KRAS^Q61H^ is the pattern of metastatic spread. While peritoneal metastasis is rare in lung cancer overall, several series report a disproportionate representation of mucinous, KRAS-mutant LUAD among cases with peritoneal carcinomatosis (10). This mirrors PDAC and CRC, where KRAS^Q61H^ is strongly associated with peritoneal and serosal dissemination, often accompanied by ascites and rapid clinical decline (8, 9). The aggressive nature of peritoneal metastases in these settings is reflected by their resistance to systemic therapy and their association with shortened survival. Together, these findings suggest that KRAS^Q61H^ defines a high-risk metastatic phenotype characterized by enhanced invasive and survival capacity within serosal environments. Importantly, these clinicopathologic features provide a clinical framework for understanding the distinctive molecular signaling properties associated with KRAS^Q61H^.

## Molecular characteristics and co-mutation landscape of KRAS^Q61H^

At the molecular level, KRAS^Q61H^ exhibits biochemical properties that distinguish it from codon 12 variants across multiple adenocarcinoma types. Codon 61 mutations impair intrinsic GTP hydrolysis more profoundly than most codon 12 substitutions. As a result, KRAS^Q61H^ remains constitutively active even in the absence of upstream receptor tyrosine kinase input, a property that distinguishes it from many other KRAS alleles (12). In LUAD, as in PDAC and CRC, KRAS^Q61H^ preferentially signals through the RAF-MEK-ERK pathway via enhanced RAF dimerization, creating a strong bias toward MAPK-driven transcriptional programs (13, 14) (**Figure 1C**).

A defining feature of KRAS^Q61H^ across adenocarcinomas is its frequent co-occurrence with TP53 mutations. In LUAD, TP53 is among the most common co-altered genes in KRAS^Q61H^ tumors, a pattern that closely mirrors PDAC, where KRAS and TP53 co-mutations are nearly ubiquitous and define a particularly aggressive molecular subtype (15, 16). Experimental models suggest that TP53 loss cooperates with KRAS^Q61H^ to promote genomic instability, epithelial–mesenchymal transition, and metastatic competence, particularly to serosal surfaces such as the peritoneum (17).

Comparative analyses across tumor types indicate that the KRAS^Q61H^/TP53 co-mutant state may be more aggressive than analogous combinations involving codon 12 alleles. In CRC, for example, codon 61 KRAS mutations are enriched in tumors with TP53 alterations and are associated with higher rates of peritoneal metastasis and poorer prognosis compared with KRAS^G12^-mutant counterparts (8, 18). In LUAD, while direct evidence remains limited by sample size, similar trends are emerging, with KRAS^Q61H^/TP53 co-mutant tumors displaying more advanced stage at diagnosis and higher metastatic burden (6).

Beyond TP53, KRAS^Q61H^ LUAD frequently harbors alterations in STK11 and KEAP1, which further shape tumor biology and immune contexture (16, 19). However, the relative contribution of these co-mutations appears to differ from that observed in codon 12-driven disease. In particular, the strong intrinsic signaling of KRAS^Q61H^ may partially override upstream metabolic and oxidative stress pathways regulated by STK11 and KEAP1, reinforcing a model in which KRAS^Q61H^ acts as a dominant oncogenic driver across tissue types (12, 14).

Collectively, these clinicopathologic and molecular parallels between LUAD and other adenocarcinomas bearing KRAS^Q61H^ support the concept that this allele defines a pan-epithelial, high-risk oncogenic state. Its association with mucinous differentiation, TP53 co-mutation, and aggressive peritoneal dissemination suggests that KRAS^Q61H^ may represent a biologically distinct and particularly invasive subtype of KRAS-driven cancer, warranting focused clinical and translational investigation.

## Diagnostic considerations

Optimal management of lung adenocarcinoma increasingly depends on comprehensive molecular profiling performed as early as possible in the diagnostic pathway (1, 20). Broad next-generation sequencing (NGS) panels that include coverage of KRAS exons 2 and 3 are essential to ensure reliable detection of codon 61 alterations, including KRAS^Q61H^, which may be missed by limited hotspot assays focused primarily on codon 12 mutations (21). Early identification of KRAS^Q61H^ is particularly important given its distinct biological behavior, lack of approved allele-specific therapies, and emerging evidence of aggressive clinical features.

Equally critical is the simultaneous assessment of co-occurring genomic alterations. NGS enables the detection of frequent co-mutations in TP53, STK11, and KEAP1, which have well-established prognostic and predictive implications in KRAS-mutant LUAD and strongly influence responses to immune checkpoint inhibitors (16, 19, 22). Delayed or incomplete molecular testing may therefore lead to suboptimal treatment selection and missed opportunities for clinical trial enrollment, particularly for patients with rare KRAS alleles.

Plasma-based circulating tumor DNA (ctDNA) analysis represents a valuable complementary approach, especially when tissue is limited or insufficient for broad sequencing. Liquid biopsy has demonstrated high concordance with tissue-based NGS for KRAS mutations. It also allows for rapid molecular characterization at diagnosis and longitudinal monitoring of clonal evolution under therapy (23, 24). Together, these diagnostic considerations underscore the importance of upfront, comprehensive NGS as a cornerstone of precision care, while directly informing therapeutic decision-making in this rare but clinically significant subgroup of LUAD.

## Therapeutic strategies

At present, there are no approved targeted therapies for KRAS^Q61H^ LUAD, and management relies on the same regimens used for KRAS wild-type disease. Immune checkpoint inhibitors (ICIs), either as monotherapy in patients with high PD-L1 expression or in combination with platinum-based chemotherapy, form the cornerstone of first-line treatment (20). Retrospective analyses suggest that the efficacy of ICIs in KRAS-mutant LUAD is strongly influenced by the co-mutation landscape. Patients with concurrent TP53 mutations often exhibit higher response rates, whereas those with STK11 or KEAP1 co-mutations demonstrate resistance, regardless of PD-L1 status or tumor mutational burden (16, 19). For Q61H, specific data remain sparse, but these broader principles of KRAS-driven disease are likely applicable.

Chemotherapy remains a critical component of care. Pemetrexed-based doublets are commonly used in nonsquamous histologies and are also appropriate for mucinous adenocarcinoma (20). Although traditional cytotoxic regimens lack molecular specificity, they continue to provide meaningful disease control, particularly when combined with ICIs.

The search for targeted approaches in Q61H has been shaped by its distinctive biology. Given the preferential reliance on MAPK signaling, MEK and RAF inhibitors represent rational strategies. Early clinical trials of MEK inhibition in unselected KRAS-mutant NSCLC were disappointing, showing modest efficacy and significant toxicity (25). This outcome may reflect biological heterogeneity across KRAS alleles rather than uniform pathway insensitivity. However, these studies grouped all KRAS alleles together, potentially obscuring activity in biologically distinct subgroups such as Q61H. Revisiting MEK or RAF inhibition in allele-specific contexts may therefore be warranted.

By contrast, strategies that hinge on SHP2 inhibition appear less promising for Q61H. Because this allele is relatively independent of upstream signaling, SHP2 blockade fails to achieve the degree of pathway suppression observed in KRAS^Q61H^-driven models (14). Nonetheless, combination regimens integrating SHP2 inhibitors with MAPK pathway blockade remain under investigation, and whether such approaches can overcome primary resistance in Q61H remains an open question.

The most exciting frontier lies in the development of pan-KRAS inhibitors and RAS-ON inhibitors. These agents, which bind active KRAS irrespective of codon, have entered early-phase clinical trials and are already showing encouraging signals across diverse RAS-mutant tumors (26). Although allele-specific activity data remain limited, the mechanistic design of these drugs suggests they should cover Q61H as effectively as other variants. If successful, they would represent the first direct targeted therapies available to this subset. Beyond small molecules, experimental strategies such as KRAS degraders and synthetic lethality approaches are also advancing, offering additional avenues for Q61H-directed therapy (27).

Taken together, these therapeutic approaches underscore both the challenges and opportunities inherent to targeting KRAS^Q61H^-mutant lung adenocarcinoma. While standard-of-care chemotherapy and immune checkpoint inhibition remain the clinical backbone, emerging targeted strategies aimed at the MAPK pathway, upstream signaling modulators, and pan-KRAS inhibition offer potential avenues for improved disease control. The distinct signaling properties of the KRAS^Q61H^ allele, including its reduced dependence on upstream regulators, highlight the need for rational combination approaches and allele-aware clinical trial design. An overview of the principal signaling dependencies, therapeutic vulnerabilities, and investigational strategies relevant to KRAS^Q61H^ LUAD is summarized schematically in **Figure 1C**, while **Table 1** provides a comparative overview of current and emerging therapeutic modalities, their clinical status, and allele-specific considerations.

**Table 1:** Therapeutic strategies for KRAS-Q61H lung adenocarcinoma.

Therapy	Evidence and relevance	Status/Key reference
Immune checkpoint inhibitors (ICIs)	Efficacy influenced by co-mutations; form the immunotherapy backbone in KRAS-mutant NSCLC. Responses enhanced by TP53 co-mutation and reduced by STK11/KEAP1 alterations.	Approved (16)
Chemotherapy	Pemetrexed-based doublets remain standard in advanced nonsquamous NSCLC and serve as the foundation for combination immunotherapy.	Approved (30)
MEK/RAF/ERK inhibitors	Target the MAPK axis hyperactivated in KRAS-Q61H; preclinical data suggest potential sensitivity, though single-agent efficacy has been modest. Combination strategies are under active study.	Investigational (25)
PI3K/AKT/mTOR inhibitors	Target a secondary signaling arm downstream of KRAS; monotherapy efficacy limited, but dual inhibition with MEK or SHP2 blockade shows synergistic effects in preclinical models.	Investigational (31)
SHP2 inhibitors	Block upstream RTK–RAS signaling; KRAS-Q61H exhibits relative resistance due to reduced SHP2 dependence. Combination approaches with MEK or RAF blockade are under investigation.	Investigational (14)
SOS1 inhibitors	Inhibit guanine-nucleotide exchange on RAS; early-phase studies show synergy with MEK inhibitors, but activity in KRAS-Q61H is yet unclear.	Investigational (32)
Pan-KRAS inhibitors/RAS degraders	Broad-spectrum inhibitors (e.g., RMC-6236) and degraders target multiple KRAS alleles, including Q61H; represent next-generation RAS-directed therapies.	Investigational (33)

## Discussion and future directions

KRAS^Q61H^ lung adenocarcinoma represents a rare but biologically and clinically meaningful subset of NSCLC. Unlike the extensively studied KRAS^G12C^ allele, Q61H lacks approved allele-specific targeted therapies and displays distinct signaling properties that shape both disease behavior and therapeutic vulnerability (3, 13, 14). Accumulating evidence suggests that Q61H-driven tumors exhibit more aggressive clinical features than other KRAS-mutant LUADs, including a tendency toward advanced-stage presentation and atypical metastatic patterns (6). The enrichment of Q61H in invasive mucinous adenocarcinoma and its association with peritoneal dissemination highlight parallels with pancreatic and colorectal adenocarcinomas, where codon 61 mutations are linked to early serosal spread and poor prognosis (7, 10). These cross-tumor similarities support the concept of KRAS^Q61H^ as a pan-epithelial, high-risk oncogenic state rather than a purely lung-restricted phenomenon.

At the molecular level, the frequent co-occurrence of TP53 mutations appears to be a defining feature of Q61H-driven disease across adenocarcinomas. Experimental and clinical data suggest that loss of TP53 function cooperates with sustained MAPK signaling to promote genomic instability, invasion, and metastatic competence, potentially explaining the aggressive behavior observed in Q61H-mutant tumors (17, 28). In LUAD, this co-mutational context also intersects with immune regulation, as TP53, STK11, and KEAP1 alterations collectively shape responsiveness to immune checkpoint blockade (16, 19). These findings underscore the importance of comprehensive genomic profiling not only to identify KRAS^Q61H^ itself, but also to define the broader molecular landscape that informs prognosis and treatment selection.

From a translational perspective, future progress will likely depend on several converging strategies aimed at overcoming the current lack of allele-specific evidence for KRAS^Q61H^. First, allele-resolved clinical trial designs that explicitly include non-G12 KRAS variants are urgently needed. Such studies could take the form of basket trials enrolling KRAS^Q61H^-mutant tumors across tissue types, or lung cancer-specific trials stratifying patients by KRAS allele and co-mutation status. These approaches would enable a more precise assessment of whether Q61H-mutant tumors derive differential benefit from MAPK pathway inhibition, pan-KRAS inhibitors, or rational combination strategies targeting parallel signaling nodes such as PI3K/AKT/mTOR, particularly in the context of frequent TP53, STK11 or KEAP1 co-alterations (25–27, 29). Importantly, integrating correlative molecular analyses into such trials may help define predictive biomarkers of response and resistance specific to the Q61H signaling state.

Second, routine upfront next-generation sequencing (NGS), coupled with the longitudinal use of circulating tumor DNA (ctDNA) analyses, will be critical for the early identification of KRAS^Q61H^ disease and for tracking clonal dynamics under therapeutic pressure. ctDNA-based approaches may facilitate real-time monitoring of emerging resistance mechanisms, enable adaptive treatment strategies, and improve patient selection for biomarker-driven trials, particularly in the metastatic setting where tissue availability is often limited (20, 23). Incorporation of liquid biopsy endpoints into prospective studies may therefore accelerate translational insights for this rare molecular subgroup.

Finally, systematic investigation of metastatic patterns, with particular attention to peritoneal dissemination, represents an underexplored but potentially high-yield research direction. Prospective characterization of metastatic routes, organ tropism, and associated molecular features may inform tailored surveillance strategies and reveal context-specific therapeutic vulnerabilities, including opportunities for intensified local or regional interventions. Together, these coordinated translational efforts may transform KRAS^Q61H^ LUAD from an understudied rarity into a biologically defined molecular subtype with evidence-based, personalized management approaches.

## References

[1] Canon J Rex K Saiki AY Mohr C Cooke K Bagal D . The clinical KRAS(G12C) inhibitor AMG 510 drives anti-tumour immunity. Nature. (2019) 575:217–23. doi: 10.1038/s41586-019-1694-1, PMID: 31666701

[2] Dogan S Shen R Ang DC Johnson ML D’Angelo SP Paik PK . Molecular epidemiology of EGFR and KRAS mutations in 3,026 lung adenocarcinomas: higher susceptibility of women to smoking-related KRAS-mutant cancers. Clin Cancer Res. (2012) 18:6169–77. doi: 10.1158/1078-0432.CCR-11-3265, PMID: 23014527 PMC3500422

[3] Skoulidis F Li BT Dy GK Price TJ Falchook GS Wolf J . Sotorasib for lung cancers with KRAS p.G12C mutation. N Engl J Med. (2021) 384:2371–81. doi: 10.1056/NEJMoa2103695, PMID: 34096690 PMC9116274

[4] Yang Y Zhang J Chen Y Xu Y Yang L Ma L . The relationship between different subtypes of KRAS and PD-L1 & tumor mutation burden (TMB) based on next-generation sequencing (NGS) detection in Chinese lung cancer patients. Transl Lung Cancer Res. (2022) 11:213–23. doi: 10.21037/tlcr-22-88, PMID: 35280306 PMC8902092

[5] Prior IA Hood FE Hartley JL . The frequency of ras mutations in cancer. Cancer Res. (2020) 80:2969–74. doi: 10.1158/0008-5472.CAN-19-3682, PMID: 32209560 PMC7367715

[6] Cooper AJ Sequist LV Lin JJ Heist RS Piotrowska Z Shaw AT . Clinicopathologic characteristics and outcomes for patients with KRAS G12D-mutant NSCLC. JTO Clin Res Rep. (2022) 3:100390. doi: 10.1016/j.jtocrr.2022.100390, PMID: 36118132 PMC9471201

[7] Der CJ Finkel T Cooper GM . Biological and biochemical properties of human rasH genes mutated at codon 61. Cell. (1986) 44:167–76. doi: 10.1016/0092-8674(86)90495-2, PMID: 3510078

[8] Serebriiskii IG Connelly C Frampton GM Newberg J Cooke M Miller V . Comprehensive characterization of RAS mutations in colon and rectal cancers in old and young patients. Nat Commun. (2019) 10:3722. doi: 10.1038/s41467-019-11530-0, PMID: 31427573 PMC6700103

[9] Bournet B Muscari F Buscail C Assenat E Barthet M Lesavre N . KRAS G12D mutation subtype is A prognostic factor for advanced pancreatic adenocarcinoma. Clin Transl Gastroenterol. (2016) 7:e157. doi: 10.1038/ctg.2016.18, PMID: 27010960 PMC4822095

[10] Shim HS Kenudson M Zheng Z Liebers M Cha YJ Hoang Hoang NT . Unique genetic and survival characteristics of invasive mucinous adenocarcinoma of the lung. J Thorac Oncol. (2015) 10:1156–62. doi: 10.1097/JTO.0000000000000579, PMID: 26200269

[11] Ichinokawa H Ishii G Nagai K Kawase A Yoshida J Nishimura M . Distinct clinicopathologic characteristics of lung mucinous adenocarcinoma with KRAS mutation. Hum Pathol. (2013) 44:2636–42. doi: 10.1016/j.humpath.2013.05.026, PMID: 24119562

[12] Gibbs JB Sigal IS Poe M Scolnick EM . Intrinsic GTPase activity distinguishes normal and oncogenic ras p21 molecules. Proc Natl Acad Sci U.S.A. (1984) 81:5704–8. doi: 10.1073/pnas.81.18.5704, PMID: 6148751 PMC391779

[13] Zhou ZW Ambrogio C Bera AK Li Q Li X Li L . KRAS(Q61H) preferentially signals through MAPK in a RAF dimer-dependent manner in non-small cell lung cancer. Cancer Res. (2020) 80:3719–31. doi: 10.1158/0008-5472.CAN-20-0448, PMID: 32605999

[14] Gebregiworgis T Kano Y St-Germain J Radulovich N Udaskin M Minden MD . The Q61H mutation decouples KRAS from upstream regulation and renders cancer cells resistant to SHP2 inhibitors. Nat Commun. (2021) 12:6274. doi: 10.1038/s41467-021-26526-y, PMID: 34725361 PMC8560773

[15] Bailey P Chang DK Nones K Johns AL Patch AM Gingras MC . Genomic analyses identify molecular subtypes of pancreatic cancer. Nature. (2016) 531:47–52. doi: 10.1038/nature16965, PMID: 26909576

[16] Skoulidis F Goldberg ME Greenawalt DM Hellmann MD Awad MM Gainor JF . STK11/LKB1 mutations and PD-1 inhibitor resistance in KRAS-mutant lung adenocarcinoma. Cancer Discov. (2018) 8:822–35. doi: 10.1158/2159-8290.CD-18-0099, PMID: 29773717 PMC6030433

[17] Morton JP Timpson P Karim SA Ridgway RA Athineos D Doyle B . Mutant p53 drives metastasis and overcomes growth arrest/senescence in pancreatic cancer. Proc Natl Acad Sci U.S.A. (2010) 107:246–51. doi: 10.1073/pnas.0908428107, PMID: 20018721 PMC2806749

[18] Yaeger R Chatila WK Lipsyc MD Hechtman JF Cercek A Sanchez-Vega F . Clinical sequencing defines the genomic landscape of metastatic colorectal cancer. Cancer Cell. (2018) 33:125–136.e3. doi: 10.1016/j.ccell.2017.12.004, PMID: 29316426 PMC5765991

[19] Arbour KC Jordan E Kim HR Dienstag J Yu HA Sanchez-Vega F . Effects of co-occurring genomic alterations on outcomes in patients with KRAS-mutant non-small cell lung cancer. Clin Cancer Res. (2018) 24:334–40. doi: 10.1158/1078-0432.CCR-17-1841, PMID: 29089357 PMC5771996

[20] Reck M Carbone DP Garassino M Barlesi F . Targeting KRAS in non-small-cell lung cancer: recent progress and new approaches. Ann Oncol. (2021) 32:1101–10. doi: 10.1016/j.annonc.2021.06.001, PMID: 34089836

[21] Lindeman NI Cagle PT Aisner DL Arcila ME Beasley MB Bernicker EH . Updated molecular testing guideline for the selection of lung cancer patients for treatment with targeted tyrosine kinase inhibitors: guideline from the college of american pathologists, the international association for the study of lung cancer, and the association for molecular pathology. J Thorac Oncol. (2018) 13:323–58. doi: 10.1016/j.jtho.2017.12.001, PMID: 29396253

[22] Skoulidis F Heymach JV . Co-occurring genomic alterations in non-small-cell lung cancer biology and therapy. Nat Rev Cancer. (2019) 19:495–509. doi: 10.1038/s41568-019-0179-8, PMID: 31406302 PMC7043073

[23] Illini O Fabikan H Johannes-Hochmair M Weinlinger C Krenbek D Brcic L . Characteristics and treatment outcomes in advanced-stage non-small cell lung cancer patients with a KRAS G12C mutation: A real-world study. J Clin Med. (2022) 11:4098. doi: 10.3390/jcm11144098, PMID: 35887862 PMC9324356

[24] Aggarwal C Thompson JC Black TA Katz SI Fan R Yee SS . Clinical implications of plasma-based genotyping with the delivery of personalized therapy in metastatic non-small cell lung cancer. JAMA Oncol. (2019) 5:173–80. doi: 10.1001/jamaoncol.2018.4305, PMID: 30325992 PMC6396811

[25] Jänne PA Shaw AT Pereira JR Jeannin G Vansteenkiste J Barrios C . Selumetinib plus docetaxel for KRAS-mutant advanced non-small-cell lung cancer: a randomised, multicentre, placebo-controlled, phase 2 study. Lancet Oncol. (2013) 14:38–47. doi: 10.1016/S1470-2045(12)70489-8, PMID: 23200175

[26] Jiang J Wang X He Y Li Q Shen L Liu Y . Translational and therapeutic evaluation of RAS-GTP inhibition by RMC-6236 in RAS-driven cancers. Cancer Discov. (2024) 14:994–1017. doi: 10.1158/2159-8290.CD-24-0027, PMID: 38593348 PMC11149917

[27] Punekar SR Velcheti V Neel BG Wong KK . The current state of the art and future trends in RAS-targeted cancer therapies. Nat Rev Clin Oncol. (2022) 19:637–55. doi: 10.1038/s41571-022-00671-9, PMID: 36028717 PMC9412785

[28] Levine AJ . Targeting the P53 protein for cancer therapies: the translational impact of P53 research. Cancer Res. (2022) 82:362–4. doi: 10.1158/0008-5472.CAN-21-2709, PMID: 35110395 PMC8852246

[29] Ryan MB Fece de la Cruz F Phat S Myers DT Wong E Shahzade HA . Vertical pathway inhibition overcomes adaptive feedback resistance to KRAS(G12C) inhibition. Clin Cancer Res. (2020) 26:1633–43. doi: 10.1158/1078-0432.CCR-19-3523, PMID: 31776128 PMC7124991

[30] Planchard D Popat S Kerr K Novello S Smit EF Faivre-Finn C . Metastatic non-small cell lung cancer: ESMO Clinical Practice Guidelines for diagnosis, treatment and follow-up. Ann Oncol. (2018) 29:iv192–237. doi: 10.1093/annonc/mdy275, PMID: 30285222

[31] Qiang M Zhao L Wang X Li Y Zhang J Liu H . Targeting the PI3K/AKT/mTOR pathway in lung cancer: mechanisms and therapeutic targeting. Front Pharmacol. (2025) 16:1516583. doi: 10.3389/fphar.2025.1516583, PMID: 40041495 PMC11877449

[32] Hofmann MH Gmachl M Ramharter J Savarese F Gerlach D Marszalek JR . BI-3406, a potent and selective SOS1-KRAS interaction inhibitor, is effective in KRAS-driven cancers through combined MEK inhibition. Cancer Discov. (2021) 11:142–57. doi: 10.1158/2159-8290.CD-20-0142, PMID: 32816843 PMC7892644

[33] Hallin J Bowcut V Calinisan A Briere DM Hargis L Engstrom LD . Anti-tumor efficacy of a potent and selective non-covalent KRAS(G12D) inhibitor. Nat Med. (2022) 28:2171–82. doi: 10.1038/s41591-022-02007-7, PMID: 36216931

